# Supplementary material for: Differential Influence of Soluble Dietary Fibres on Intestinal and Hepatic Carbohydrate Response
Source: Nutrients. 2021 Nov 27;13(12):4278. doi: 10.3390/nu13124278 (PMC8706546; doi:10.3390/nu13124278)
Supplement: Supplementary file 1 [file nutrients-13-04278-s001.zip › nutrients-1450210-supplementary.pdf]

Table S1. Diet Formulas and nutrient composition.

Nutrient Composition	*LF*		*LF In*		*LF Psy*		*LF In Psy*	
**%**	**Kcal**		**Kcal**		**Kcal**		**Kcal**	
Protein	20		20		20		20	
Carbohydrate	70		70		70		70	
Fat	10		10		10		10	
Diet component	**gm**	**Kcal**	**gm**	**Kcal**	**gm**	**Kcal**	**gm**	**Kcal**
Casein	200	800	200	800	200	800	200	800
L-Cystine	3	12	3	12	3	12	3	12
Corn starch	506.2	2024.8	478	1912	487.5	1950	482.8	1931
Maltodextrin 10	125	500	125	500	125	500	125	500
Sucrose	68.8	275	68.8	275	68.8	275	68.8	275
Cellulose,	50	0	50	0	50	0	50	0
Inulin	0	0	75	113	0	0	37.5	56
Psyllium	0	0	0	0	75	75	37.5	38
Lard	20	180	20	180	20	180	20	180
Soybean Oil	25	225	25	225	25	225	25	225
Mineral mix S10026	10	0	10	0	10	0	10	0
Dicalcium Phosphate	13	0	13	0	13	0	13	0
Calcium Carbonate	5.5	0	5.5	0	5.5	0	5.5	0
Potassium Citrate	16.5	0	16.5	0	16.5	0	16.5	0
Vitamin mix V10001	10	40	10	40	10	40	10	40
Choline Bitartrate	2	0	2	0	2	0	2	0
Yellow Dye #5 FD&C	0.04	0	0.025	0	0	0	0	0
Red Dye #40 FD&C	0	0	0.025	0	0.05	0	0.025	0
Blue Dye #1 FD&C	0.01	0	0	0	0	0	0.025	0
Total	1055.05	4057	1101.85	4057	1111.35	4057	1106.65	4057

Table S2. Primer sequences used for qPCR analysis.

Gene	Forward	Reverse
Acaca	GAGGAAGTTGGCTATCCAG	GCAGGAAGATTGACATCAGC
Aldob	CTGTGTTGAGGATTGCTGACCAG	TCAGGAAGCACCTCTGGCTCAA
Chrebp	GAGTGCTTGAGCCTGGCTTACA	GCTCTCCAGATGGCGTTGTTCA
Fasn	AGTGCGTGGGGCGCAATCTC	CGCTCGGCTCGATGGCTCAG
Fgf21	ACACAATTCCAGCTGCCTTG	TAGAGGCTTTGACACCCAGG
Irs1	TGTCACCCAGTGGTAGTTGCTC	CTCTCAACAGGAGGTTTGGCATG
Khk	CCCACCGCCCCGAGTAGTAGACA	CACACCTGCCGGGGAATGG
Mgam	ATGGAGAGTGACGTTGTAAACAC	GGAAGCGATTTGATGTCGGTA
Srebf1	TGACCCGGCTATTCCGTGA	CTGGGCTGAGCAATACAGTTC
Sglt1	TGGTGTACGGATCAGGTCATTG	TTCAGATAGCCACACAGGGTACA
Sis	GCTATCGCTCTTGTTGTGGTT	TTCCAGGACTAGGGGTTGAAG
Slc2a2	GTCGCCTCATTCTTTGGTG	CTGATACACTTCGTCCAGC
Slc2a5	TCATGACCATCCTCACGATCTTT	GCGGCCGTCAGCACTAAG
Tbp	GAAGCTGCGGTACAATTCCAG	CCCCTTGTACCCTTCACCAAT

Table S3. Full correlation analysis results.

Phylum	Genus	Metabolic class	metabolite	R	p-value
**Proteobacteria**	Parasutterella	Azoles	Urocanate	-0.903	3.40E-04
**Proteobacteria**	Parasutterella	Organic Acids	Succinate	0.879	8.10E-04
**Proteobacteria**	Parasutterella	Others	Isopropanol	-0.867	1.20E-03
**Proteobacteria**	Parasutterella	Fatty Acids	2-methylbutyrate	-0.842	2.20E-03
**Proteobacteria**	Parasutterella	Organic Acids	Lactate	0.794	6.10E-03
**Proteobacteria**	Parasutterella	Amino Acids	Citrulline	-0.77	9.20E-03
**Proteobacteria**	Parasutterella	Fatty Acids	Isovalerate	-0.758	0.011

**Proteobacteria**	Parasutterella	Carbohydrates	Mannose	0.758	0.011
**Proteobacteria**	Parasutterella	Amino Acids	Methionine	-0.745	0.013
**Proteobacteria**	Parasutterella	Organic Acids	Fumarate	0.721	0.019
**Proteobacteria**	Parasutterella	Others	3-Methyl-2-oxovalerate	-0.721	0.019
**Proteobacteria**	Parasutterella	Organic Acids	Taurine	0.697	0.025
**Proteobacteria**	Parasutterella	Carbohydrates	Arabinose	0.685	0.029
**Proteobacteria**	Parasutterella	Carbohydrates	Glucose	-0.673	0.033
**Proteobacteria**	Parasutterella	Alcohols	Propylene glycol	0.661	0.038
**Verrucomicrobia**	Akkermansia	Carbohydrates	Arabinose	0.927	1.10E-04
**Verrucomicrobia**	Akkermansia	Organic Acids	Fumarate	0.842	2.20E-03
**Verrucomicrobia**	Akkermansia	Others	Choline	0.818	3.80E-03
**Verrucomicrobia**	Akkermansia	Carbohydrates	Mannose	0.806	4.90E-03
**Verrucomicrobia**	Akkermansia	Others	3-Methyl-2-oxovalerate	-0.782	7.50E-03
**Verrucomicrobia**	Akkermansia	Organic Acids	Lactate	0.758	0.011
**Verrucomicrobia**	Akkermansia	Carbohydrates	Glucose	-0.733	0.016
**Verrucomicrobia**	Akkermansia	Organic Acids	Taurine	0.721	0.019
**Verrucomicrobia**	Akkermansia	Amino Acids	Citrulline	-0.709	0.022
**Verrucomicrobia**	Akkermansia	Amino Acids	Methionine	-0.709	0.022
**Verrucomicrobia**	Akkermansia	Alcohols	Propylene glycol	0.697	0.025
**Verrucomicrobia**	Akkermansia	Azoles	Urocanate	-0.685	0.029
**Verrucomicrobia**	Akkermansia	Organic Acids	Succinate	0.685	0.029
**Verrucomicrobia**	Akkermansia	Fatty Acids	Isovalerate	-0.661	0.038
**Verrucomicrobia**	Akkermansia	Others	Isopropanol	-0.636	0.048
**Actinobacteria**	Enterorhabdus	Fatty Acids	Isovalerate	0.952	2.30E-05
**Actinobacteria**	Enterorhabdus	Fatty Acids	2-methylbutyrate	0.867	1.20E-03
**Actinobacteria**	Enterorhabdus	Others	Isopropanol	0.855	1.60E-03
**Actinobacteria**	Enterorhabdus	Others	3-Methyl-2-oxovalerate	0.806	4.90E-03
**Actinobacteria**	Enterorhabdus	Organic Acids	Succinate	-0.77	9.20E-03
**Actinobacteria**	Enterorhabdus	Organic Acids	Fumarate	-0.758	0.011
**Actinobacteria**	Enterorhabdus	Carbohydrates	Glucose	0.758	0.011
**Actinobacteria**	Enterorhabdus	Amino Acids	Citrulline	0.758	0.011
**Actinobacteria**	Enterorhabdus	Amino Acids	Methionine	0.758	0.011
**Actinobacteria**	Enterorhabdus	Amino Acids	Proline	-0.758	0.011
**Actinobacteria**	Enterorhabdus	Azoles	Urocanate	0.721	0.019
**Actinobacteria**	Enterorhabdus	Organic Acids	Lactate	-0.709	0.022
**Actinobacteria**	Enterorhabdus	Amino Acids	Arginine	-0.648	0.043
**Actinobacteria**	Enterorhabdus	Fatty Acids	Butyrate	0.648	0.043
**Actinobacteria**	Enterorhabdus	Others	Choline	-0.636	0.048
**Actinobacteria**	Enterorhabdus	Carbohydrates	Arabinose	-0.636	0.048
**Proteobacteria**	Odoribacter	Organic Acids	Succinate	-0.915	2.00E-04
**Proteobacteria**	Odoribacter	Others	3-Methyl-2-oxovalerate	0.915	2.00E-04
**Proteobacteria**	Odoribacter	Fatty Acids	2-methylbutyrate	0.891	5.40E-04
**Proteobacteria**	Odoribacter	Azoles	Urocanate	0.867	1.20E-03
**Proteobacteria**	Odoribacter	Fatty Acids	Isovalerate	0.83	2.90E-03
**Proteobacteria**	Odoribacter	Amino Acids	Citrulline	0.806	4.90E-03
**Proteobacteria**	Odoribacter	Carbohydrates	Arabinose	-0.758	0.011
**Proteobacteria**	Odoribacter	Amino Acids	Methionine	0.733	0.016
**Proteobacteria**	Odoribacter	Organic Acids	Lactate	-0.733	0.016
**Proteobacteria**	Odoribacter	Alcohols	Propylene glycol	-0.721	0.019

**Proteobacteria**	Odoribacter	Others	Isopropanol	0.685	0.029
**Proteobacteria**	Odoribacter	Organic Acids	Taurine	-0.661	0.038
**Proteobacteria**	Odoribacter	Organic Acids	Fumarate	-0.648	0.043
**Proteobacteria**	Odoribacter	Others	Choline	-0.636	0.048
**Firmicutes**	Lachnoclostridium	Fatty Acids	2-methylbutyrate	0.976	1.50E-06
**Firmicutes**	Lachnoclostridium	Organic Acids	Succinate	-0.927	1.10E-04
**Firmicutes**	Lachnoclostridium	Azoles	Urocanate	0.879	8.10E-04
**Firmicutes**	Lachnoclostridium	Fatty Acids	Isovalerate	0.867	1.20E-03
**Firmicutes**	Lachnoclostridium	Others	3-Methyl-2-oxovalerate	0.842	2.20E-03
**Firmicutes**	Lachnoclostridium	Amino Acids	Methionine	0.83	2.90E-03
**Firmicutes**	Lachnoclostridium	Organic Acids	Lactate	-0.806	4.90E-03
**Firmicutes**	Lachnoclostridium	Amino Acids	Citrulline	0.77	9.20E-03
**Firmicutes**	Lachnoclostridium	Others	Isopropanol	0.721	0.019
**Firmicutes**	Lachnoclostridium	Carbohydrates	Glucose	0.721	0.019
**Firmicutes**	Lachnoclostridium	Carbohydrates	Mannose	-0.721	0.019
**Firmicutes**	Lachnoclostridium	Others	Choline	-0.709	0.022
**Firmicutes**	Lachnoclostridium	Organic Acids	Fumarate	-0.661	0.038
**Firmicutes**	Lachnoclostridium	Alcohols	Propylene glycol	-0.661	0.038
**Firmicutes**	Lachnoclostridium	Organic Acids	Taurine	-0.636	0.048
